# Identification and Characterization of Eleven Novel Human Gamma-Papillomavirus Isolates from Healthy Skin, Found at Low Frequency in a Normal Population

**DOI:** 10.1371/journal.pone.0077116

**Published:** 2013-10-14

**Authors:** Jingjing Li, YaQi Pan, QiuJu Deng, Hong Cai, Yang Ke

**Affiliations:** Key Laboratory of Carcinogenesis and Translational Research (Ministry of Education), Peking University Cancer Hospital & Institute, Beijing, China; Institut Pasteur, France

## Abstract

Eleven novel human papillomavirus (HPV) types were isolated and characterized from healthy individuals in China. HPV163 belongs to the γ-1 species, HPV 164 and HPV 168 fit in the γ-8 species, HPV 165 and KC5 belongs to the γ-12 species, HPV 168 is closely allied with the γ-4 species, HPV 169 is closely related to the γ-11 species, and HPV 170 is related to the γ-12 species. In addition, HPV 161, HPV 162, and HPV 166 may form a new HPV species of the γ-PV genus. The prevalence of these HPV types in the normal population is low.

## Introduction

Papillomavirus (PVs) are a distinct viral family, which infect the epithelia of vertebrates. Infections may be prolonged without clinical symptoms; however, they may induce neoplasia in certain sites [1,2]. PV infection is species restricted and is rarely transmitted across species. Human papillomavirus is one of the most important groups of the PV family, and it can be divided roughly into two tropism groups, cutaneotropic and mucosotropic HPV types, which infect skin and mucosal tissues respectively. Because lack of effective *in vivo* PV culture technology and PVs do not naturally induce robust antibody production in host, traditional virus classification is not appropriate for PVs. A typical novel PV type is identified and defined by cloning full-length PV genome whose L1 gene nucleotides sequences is at least 10% dissimilar from any other known PV type. The classification of PV types is based on construction of a phylogenetic tree using the L1 genes [3]. Within the *papillomaviridae*, 37 separate genera have been established, and five of them infect human beings. Five human papillomavirus genera including Alpha, Beta, Gamma, Mu, and Nu contain at least 150 HPV types that have been fully characterized. However, it is believed that there is potentially much greater number of unknown HPV types to be identified, and most of the undiscovered HPVs are expected to be cutaneotropic types. 

Compared with mucosotropic HPV types, cutaneotropic HPV types exhibited very different characteristics not only in their nucleotide sequences but also in their biologic behavior. It is well known that approximately 13 high-risk mucosotropic HPV types can cause cervical cancer (IARC monographs), but none of the cutaneotropic HPV types has been confirmed to be associated with any malignant skin disease [4], which indicates that the cutaneotropic HPVs may exhibit lower pathogenicity. The prevalence and diversity of the cutaneotropic HPV group are both much higher than mucosotropic HPVs in a healthy population [5-7]. In addition, normal human skin harbors an array of papillomaviruses, and most of them are unknown previously [8]. Therefore there is great potential for discovery of novel HPV types from the normal skin of healthy individuals. Isolation and characterization novel HPV genomes will reveal the existence of distinct human papillomavirus (HPV) types and will provide basic information for understanding the biologic significance and the basis of HPV evolution. Many studies focus on identifying novel HPV from common warts, keratotic lesions or cutaneous squamous cell carcinoma [9-14], but few studies have investigated novel HPV types originated from healthy individuals. 

In this study, full-length genomes of eleven novel HPV types were isolated, fully cloned, sequenced and characterized from healthy individuals in rural Anyang, Henan province, China. All of these eleven novel HPVs belong to the Gamma-papillomavirus genus, and show very low prevalence in this population. 

## Materials and Methods

### Skin samples

Exfoliated skin cell samples used in this study were collected from individuals who participated in a perspective cohort study of esophageal cancer launched in rural An Yang, China [15]. For each individual, exfoliated cells were collected by scraping the palms of the hands using glass slides and then collecting the resultants material into 1 ml of 0.9% sodium chloride. Cells were centrifuged at 10,000 rpm for 10 min. Precipitates were collected and stored in -80°C before use. DNA was extracted using the spin-column method (Tiagen, a subsidiary of Qiagen in China), and was stored at -20°C before analyzed. 

### Isolation and characterization of novel HPV types

Novel HPV clones were initially identified by PCR amplification with FAP6085/64 primer pairs [16,17] producing a 377 bp L1 fragment. A total of 149 putative novel HPV types were identified in detecting skin HPV infection from a healthy population [18], and 11 of them were successfully whole genome sequenced and cloned into vectors. 

Rolling circle amplification (RCA) was performed to amplify the circle virus genome DNA by using illustra TempliPhi 100 Amplification Kit (GE Healthcare, Amersham, UK), as previous described [19]. RCA products were diluted 20-100 fold as a PCR template. Type-specific primers were designed for each putative novel HPV types based on the FA amplified fragment sequences. Reverse PCR was performed using the NEB LongAmp *Taq* system (New England Biolabs). The 30 µl PCR reaction buffer consisted of 0.4 µM of each primer, 200 µM of each dNTP, 1x LongAmp buffer, 2.5 mM Mg_2_SO_4_ and 2.5 U LongAmp *Taq* enzyme, and 1 µl of diluted RCA product template. PCR was initiated by a preheating step at 94°C for 1 min, followed by 40 cycles consisting of 94 °C for 20 s, 60°C for 40 s, and 65 °C for 7 min. The final elongation step was at 65 °C for 10 min. The PCR amplicons were excised from an agarose gel and cloned into a pCR 2.1 TOPO TA vector (Invitrogen), according to the manufacturers’ instructions, and sequenced with M13 primers.

**Figure 1:**
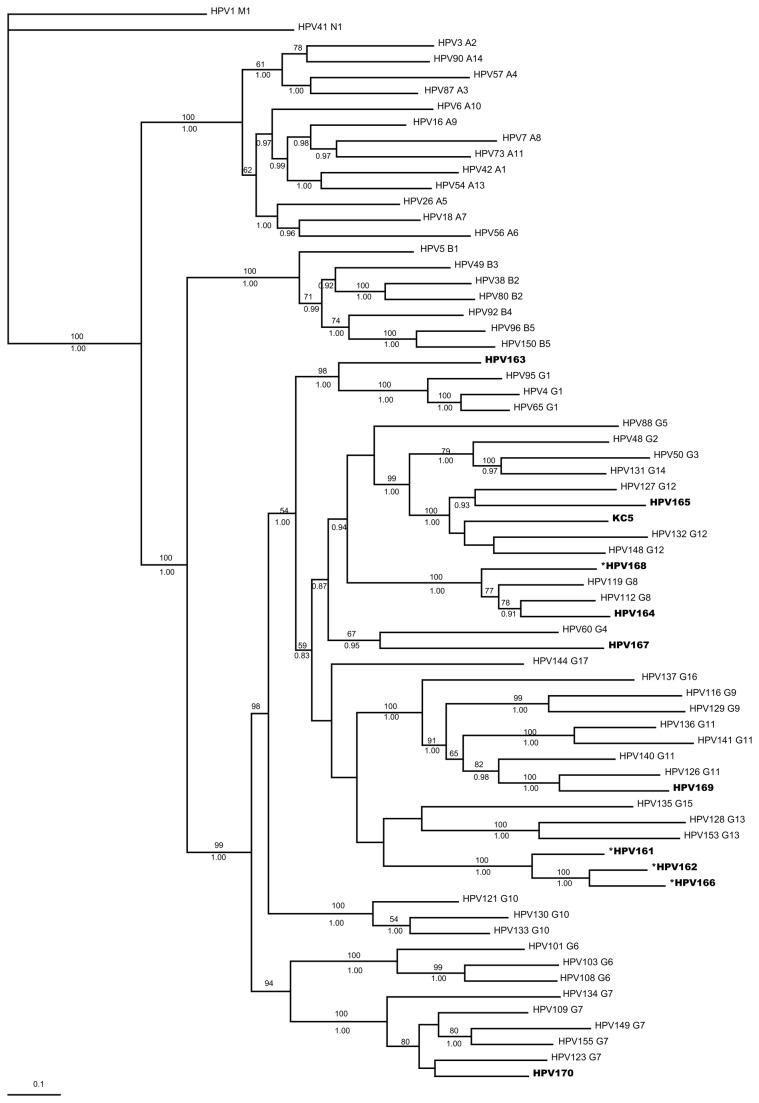
Systematic positions of novel HPVs. Eleven novel HPVs (HPV 161-170, KC5) were nested within the *Gammapapillomavirus* Genus. The evolutionary history was inferred by using the Bayesian and ML methods. A Bayesian consensus tree was based on the whole length of the L1 ORFs. Numbers on branches are bootstrap support values to the clusters to their right (upper number: ML criterion, values, 50 are not shown) and Bayesian posterior probabilities (lower number, values, 0.90 are not shown). The tree is rooted with selected Mu and Nu species. The analysis involved 68 nucleotide sequences. All positions containing gaps and missing data were eliminated. There were a total of 1263 positions in the final dataset. Bar, 0.2 nucleotide substitutions per site. *: Particular types show incongruent systematic positions as inferred from E1-E2 gene phylogenies (Supplementary file).

To construct HPV clones containing whole genome sized fragment, other sets of type-specific primers were designed by using Primer3 software [20]. With this design, each HPV gene remained intact except in the LCR region (Figure 1). For HPV 161-166, HPV 169, HPV 170, and KC5, type-specific long-template PCR was performed using the NEB LongAmp *Taq* system (New England Biolabs) with the same reaction system and conditions as described above, with primers as listed in Table S1. For Cloning HPV 167 and HPV 168, two overlap fragments were amplified by two pairs of primers (Table S2). PCR amplifying overlap fragments were performed in a 30 µl reaction buffer with 0.5µM primers, 200µM each of dNTP, 1x Phusion HF buffer, 0.5 U Phusion DNA polymerase (New England Biolabs), and 1 µl of diluted RCA product as a template. The conditions for high-fidelity PCR were: 98°C for 30s, and then 35 cycles at 98°C for 10s, 60°C for 15s, and 72°C for 4 min. The final extension was performed at 72°C for 10 min. Blunted PCR products were cloned by using the CloneJET^TM^ PCR Cloning Kit (Fermentas) according to the manufacturer’s instructions. Full sequencing by primer walking was carried out for all plasmid clones containing whole HPV genomes. Plasmids clones and sequences were submitted to the Reference Centre for Papillomaviruses, Heidelberg, Germany for official consideration. The complete genome sequences were submitted to the NCBI/GenBank database. 

### Protein Prediction

ORFs were predicted by using the ORFinder (http://www.ncbi.nlm.nih.gov/gorf/gorf.ht-ml). E4 genes were identified by searching the proline-rich region (private communication with Dr. de Villiers, Dr. Eric Schulz, and Dr. Boštjan Kocjan) and confirmed by manual alignment of nucleotide and amino acid sequences to the homologous regions of related HPV types. Further genome structure analysis was predicted by directly searching the expected conserved protein or nucleotide sequences [21] with assistance of the CLC sequence view 6 program (http://www.clcbio.com/products/clc-sequence-viewer/). 

### Sequence alignment and Phylogenetic analysis

Multiple alignment of full-length L1 ORF of 11 novel HPV and 57 previously characterized HPV types obtained from the PaVE database (http://pave.niaid.nih.gov/ #home) was performed by using the MEGA5 software package [22]. Fifty-seven previously knew HPVs include 35 published sequences of the gamma genus, and 22 representative HPV types for the species of Alpha and Beta genera. Full-lengths of E1-E2 ORF concatenations were also used in this study to explore possible incongruent tree topologies. 

Models of nucleotide substitution were selected by the jModel Test version 3.7 [23], and the GTR+I+G substitution model was the best-fit model for both maximum likelihood and Bayesian analysis methods. ML phylogenetic trees were constructed by MEGA5 and data were bootstrap-resampled 1000 times to determine node support for the best trees. Bayesian phylogenetic analyses were performed with the MrBayes version 3.1.2 [24].The phylogenetic tree was displayed by using the Treeview [25].

PASC (Pairwise Sequence Comparison) is a web tool for analysis of pairwise identity distribution within viral families. The identities are pre-computed for every pair within the families with distribution plotted in the form of histogram where each bar corresponds to an interval of identities [26]. The global alignments function of this program was used to calculate the pairwise identities of these novel HPV types within the *Papillomaviridae.*

### Detection of novel HPV types among normal population

FAP6085/64 primer pairs were used to detect a broad spectrum of cutaneous HPV among a healthy population with no symptoms of any skin disease. A total of 2210 skin exfoliated cell samples were collected. 25 µl of reaction solution contained 5 µl DNA, 0.75 µM of each FAP6085 (5’-CCWGATCCHAATMRRTTTGC-3’) and 64 (5’-CCWATATCWVHCATITCICCATC-3’) primer, 200 µM each of dNTP (Fermentas), 3.5mM MgCl_2_, 0.625 unit Hotstar Taq polymerase (Qiagen), and Hotstar reaction buffer. The PCR reaction conditions were: 95°C for 5 min, followed by 45 cycles of 50s at 94°C, 50s min at 49 °C and 50s at 72°C. Sterile water was used to replace DNA for the NTC sample (negative template control), and 1000 copies of HPV 8 plasmid served as a positive control. 

### Ethics Statement

None of the 2210 samples from the Anyang population were collected solely for the purpose of this study. Samples of exfoliated skin cells were collected prospectively in our previous studies. All samples included in the study were collected in compliance with the Helsinki Declaration. This study was approved by the institutional review boards of Beijing Cancer Hospital. All participants gave written informed consent for use of their samples in research. In order to protect the rights of the participants, all samples used in the study were coded and tested anonymously.

## Results

The clones and corresponding complete sequences were submitted to the International Reference Centre for Papillomaviruses at the German Cancer Research Centre, Heidelberg, Germany. The novel HPV types were designated HPV 161 (JX413109), HPV 162 (JX413108), HPV 163 (JX413107), HPV 164 (JX413106), HPV 165 (JX444072), HPV 166 (JX413104), HPV 168 (KC862317), HPV 167 (KC862318), HPV 169 (JX413105), and HPV 170 (JX413110). HPV Clone for KC5 (JX444073) degraded in transit and could not be reproduced in the lab, so it could not be validated by the Reference Centre.

### Genome organization of the eleven novel HPV types

The genome lengths of these 11 novel HPVs are 7128bp to 7415bp, and the genomic G+C content is between 35.83% (HPV 167) and 38.66% (HPV 166). They all have a genomic organization in common as has been reported in cutaneous PVs [10,12]. Each contains seven ORFs, which potentially encode five early genes E6, E7, E1, E2, E4, and two late genes L2 and L1 (Table 1). There are no nucleotide sequences for the E5 gene between early and late genes and such sequences are typically missing in β-PV and γ-PV [13]. The putative E4 ORFs of HPV 161, 162, 163, 165, 166, 167, and 170 do not have start codons, however sequences that may potentially encode this gene are present in these genomes, similar to E1 ORFs described previously [21]. Further validation of E4 transcripts should be carried out. 

**Table 1:** Genome lengths, nucleotide positions on the genome and sizes of individual ORFs and URRs.

**HPV type**	**Genome length Nucleotide (nt) position, size (aa) of ORF and URR region**								
	Genome	E6	E7	E1	E2	E4	L2	L1	#URR
HPV 161	7237	199-621 (141)	618-911 (98)	895-2709 (605)	2642-3820(393)	3237-3578 (342)*	3820-5361 (514)	5373-6896 (508)	539
HPV 162	7212	127-546 (140)	546-851 (102)	835-2658 (608)	2591-3760 (390)	*3096-3518 (141)	3757-5277 (507)	5288-6808 (507)	530
HPV 163	7231	177-605 (143)	602-901 (100)	888-2711 (608)	2647-3867 (407)	*3236-3628 (131)	3870-5420 (517)	5435-6958 (508)	449
HPV 164	7231	89-508 (140)	505-798 (98)	782-2584 (601)	2514-3701 (396)	2986-3477 (164)	3703-5277 (525)	5289-6866 (526)	453
HPV 165	7128	96-515 (140)	512-793 (94)	777-2603 (609)	2536-3726 (397)	*3110-3481 (124)	3801-5243 (481)	5256-6776 (507)	447
HPV 166	7212	†327-716 (140)	718-1023 (102)	1007-2830 (608)	2763-3932 (390)	*3358-3690 (111)	3932-5464 (511)	5475-6995(507)	516
HPV 167	7228	96-515 (140)	512-805 (98)	789-2603 (605)	2536-3741 (402)	*3128-3499 (124)	3750-5258 (503)	5269-6819 (517)	504
HPV 168	7204	381-800 (140)	797-1090 (98)	1074-2876 (601)	2806-3984 (393)	3278-3760 (161)	3986-5548 (521)	5560-71313 (524)	453
HPV 169	7251	157-588 (144)	591-890 (100)	874-2685 (604)	2621-3793 (391)	3048-3566 (173)	3795-5285 (497)	5294-6871 (526)	536
HPV 170	7415	316-753 (146)	729-1013 (95)	1003-2856 (618)	2798-4012 (405)	*3363-3770 (136)	4014-5582 (523)	5593-7149 (519)	581
KC5	7143	80-502 (141)	492-773 (94)	757-2559 (601)	2477-3664 (396)	3075-3434 (120)	3666-5201 (512)	5212-6732 (507)	489

# Upstream regulatory region; * Putative E4 ORF without an ATG; † E6 uses the second ATG.

*In silico analysis* shows that the E6 ORF of all these novel HPV types contains two zinc-binding domains CxxC(x)_28–30_-CxxC, separated by 36 amino acids. The E7 ORF contains one zinc-binding domain [CxxC(x)_29–30_-CxxC], and six HPVs (HPV 161, HPV 162, HPV 165, HPV 166, and HPV 167) have the LxCxE motif for binding to the pRB protein, but the remainder of these novel HPV types lack this motif. The ATP binding site of the ATP-dependent helicase (GPPDTGKS) is conserved in the carboxy-terminal region of E1 in HPV 161, HPV 162, HPV 163, HPV 165, HPV 166, HPV 168, and KC5. The ATP binding site is modified slightly in HPV 164 and HPV 167 as “GPPD**S**GKS”, in HPV 169 as “G**V**PDSGKS”, and in HPV 170 as “GPP**N**TGKS” (the amino acid in bold indicate mutation site). The length of the upstream regulatory region (URR) is from 447 bp (HPV 165) to 581 bp (HPV 170) (Table 1), similar to the size of URRs in other HPV types of the genus Gamma-papillomavirus [9,12]. Within the URR region of all these novel HPV types, putative E2-binding sites (ACCN_6_GGT) were identified (Table S1). 

As determined using the PASC global alignment analysis searching for the best matches within the papillomavirus family (http://www.ncbi.nlm.nih.gov/sutils/pasc/viridty.cgi?cmdresult=main&id=347), HPV 161 and HPV 162 are both most related (67%) to HPV 135, HPV 163 share 73% nucleotide sequence identity with the whole genome of HPV 95, the sequence of HPV 164 is 78% identical to HPV 112, HPV 165 is 70% identical to HPV 148, HPV 166 is 66% identical to HPV 130, HPV 167 is 76% identical to HPV 112, HPV 168 is 67% identical to HPV 60, HPV 169 is 74% identical to HPV 126, HPV 170 is 76% identical to HPV 123, and the KC5 is most related (72%) to HPV 148. L1 nucleotide sequence similarities among these novel HPV types and closest known HPVs are listed in Table 2. The criteria for definition of a new PV types is less than 90% similarity in the L1 nucleotide sequence [1,3] with any known HPV type, and current data confirm all elven HPV isolates in this study are new HPV types. 

**Table 2:** L1 nucleotide sequence similarities between novel HPV types and known HPVs by the Blast program (Ling et al., 2013).

**HPV type**	**GenBank accession numbers**	**Most related novel HPV types**	**Max Identity**	**Most related known HPV types (species)**	**Max Identity**
HPV 161	JX413109	*HPV 162	78%	HPV 135 (γ-15)	67%
HPV 162	JX413108	*HPV 166	82%	HPV 135 (γ-15)	67%
HPV 163	JX413107	HPV 170	68%	*HPV 95 (γ-1)	73%
HPV 164	JX413106	KC5	67%	*HPV 112 (γ-8)	78%
HPV 165	JX444072	*KC5	70%	*HPV 148 (γ-12)	70%
HPV 166	JX413104	*HPV 162	82%	HPV 130 (γ-10)	66%
HPV 167	KC862317	HPV 170	66%	*HPV 60 (γ-4)	67%
HPV 168	KC862318	*HPV 164	76%	*HPV 112 (γ-8)	76%
HPV 169	JX413105	KC5	65%	*HPV 126 (γ-11)	74%
HPV 170	JX413110	HPV 163	68%	*HPV 123 (γ-7)	76%
KC5	JX444073	HPV 165	70%	*HPV 148 (γ-12)	72%

* Most closely related HPV types

### Phylogenetic representation of novel HPV types in relation to known Human Papillomavirus

**Figure 2:**
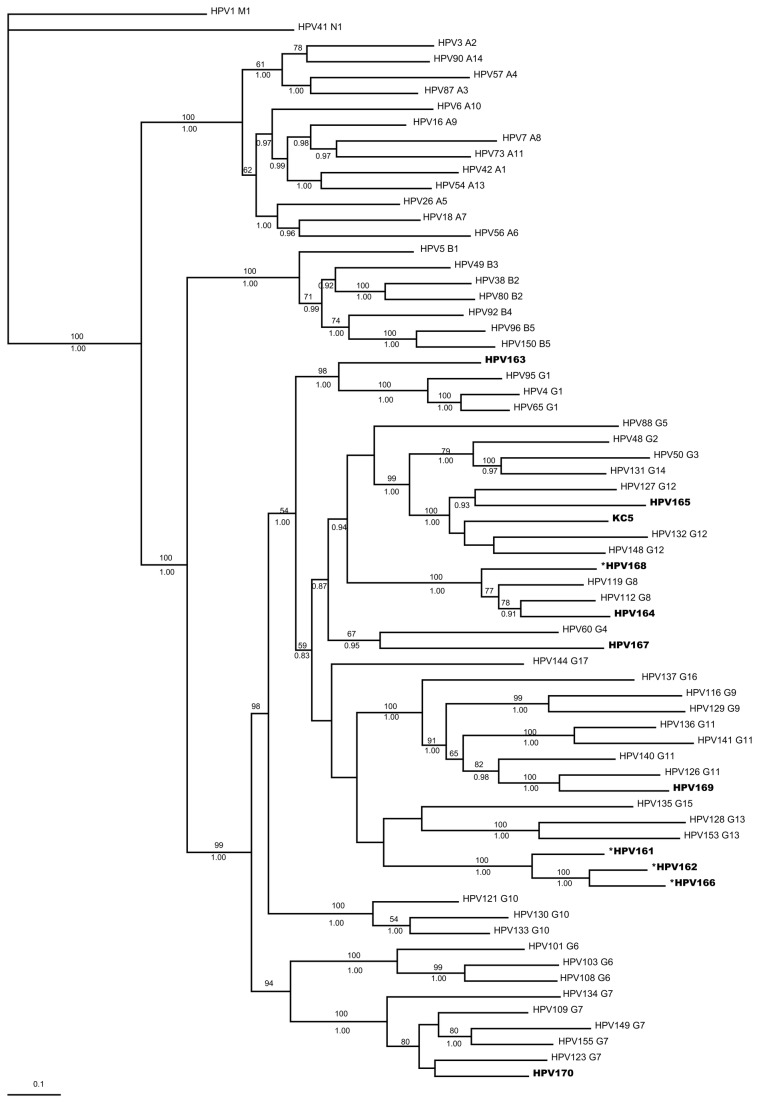
Strategy of amplification of the intact novel HPV genome. (A) Reverse PCR primers were design according to the known FA fragment nested in the L1 ORF to amplify the almost intact HPV genome, but lack of a small part of the genome; (B) Reverse PCR primers were designed according to the sequence obtained from (A), and primers were located inside the LCR region.

Phylogenetic trees were produced using either the maximum-likelihood or Bayesian method. As very similar topologies were observed, we therefore combined the ML results and Bayesian results for the L1 tree and the E1-E2 tree, respectively (Figure S1-S4). Phylogenetic analysis based on both the L1 gene and E1-E2 genes indicates that these 11 novel HPV types cluster into the Gamma genus, although there are topological inconsistences between early-gene- and late-gene-derived phylogenies among several novel HPV types. Based on L1 ORF sequences, these 11 novel HPVs were classified into flowing species, as follows: HPV 163 to the γ-1 species; HPV 164 and HPV 168 to the γ-8 species; HPV 165 and KC5 to the γ-12 species; HPV 168 to the γ-4 species; HPV 169 to the γ-11 species; and HPV 170 to the γ-12 species. It is noteworthy that HPV 161, HPV 162, and HPV 166 may form a new monophyletic group, whose closest relationship is with the γ-15 species (Figure 2). 

In the E1-E2 early-gene-derived phylogenetic analysis, HPV 161, HPV 162, HPV 166, and HPV 168 showed incongruent results compared with late-gene-derived L1 phylogenies. In the early gene analysis, HPV 161, HPV 162, and HPV 166 were monophyletic, but they clustered with the γ-12 species rather than the γ-15 species in the late gene analysis. In addition, the most closely related species of HPV 168 was γ-8 but not γ-4 based on the result of the late gene analysis.

### Prevalence of novel HPV types in the healthy population

A total of 2210 skin exfoliated cell samples were collected from the Anyang rural population and tested for cutaneous HPV infection. The prevalence of novel HPV types was extremely low. The infection of novel HPV types appeared either alone or in combination with other HPV types (Table 3).

**Table 3:** Characteristics of novel HPV positive samples and co-infection information.

**Type**	**No**	Gender*	**Age**	**Presence of other HPV**	**Type**	**No**	**Gender**	**Age**	**Presence of other HPV**
HPV 161	7	M	50	HPV 5, KC94	HPV 167	8	F	53	-
		M	42	HPV 21			F	35	FA48
		F	29	-			M	31	-
		F	41	FA12.2, HPV 3			M	54	FA57, HPV 10, KC139
		F	36				M	47	HPV 16
		M	32	HPV 75			F	53	HPV 3
		M	49	HPV 5, KC73			F	31	FA105, HPV 11,HPV 47
HPV 162	5	F	45	-			M	38	-
		M	58	-	HPV 168	2	M	45	HPV 3
		M	61	HPV 3			M	46	FA54, HPV 3
		M	32	-	HPV 169	1	F	51	HPV 94
		F	45	HPV 3	HPV 170	9	M	45	-
HPV 163	2	F	34	-			F	56	-
				HPV 92			F	37	KC 34
HPV 164	4	M	34	HPV 12, HPV 3			M	32	-
		M	45	-			M	56	HPV 3
		F	32	-			F	63	-
		M	37	HPV 57			M	54	HPV 3
HPV 165	2	F	27	-			F	66	-
		F	38	-			F	53	-
HPV 166	8	F	41	HPV 22	KC5	1	F	44	HPV 22, HPV 94, KC 5
		M	54	HPV 27, HPV 49					
		M	48	-					
		M	35	-					
		M	62	HPV 47					
		F	43	-					
		M	57	-					
		F	43	HPV 3					

* “M”: Male; “F”: Female; “- ”: No other HPV infection

## Discussion

The skin serves both as a barrier to prevent microbial infection and as a habitat for a diversity of commensal and pathogenic bacteria, fungus, and virus, which contribute to both human health and disease [27,28]. Human papillomavirus are distributed widely on human skin and exhibit great genetic diversity [6,8,29]. Unlike HPVs which inhabit the genital tract, HPVs from the cutaneous ecosystem engage in much milder relationships with human beings [4]. Present evidence suggests that infective cutaneotropic HPVs may only lead to epidermodysplasia verruciformis (EV) or other benign skin diseases [30-32]. Differences in the pathogenic capacity of HPVs are closely related to variations in the viral genome. The analysis of genomes provides precision sequence changes that may be evolutionarily important and comprise biologic forces driving HPVs into discrete ecosystems.

The majority of putative HPV types have been initially isolated from healthy skin without lesions suggesting the generally commensal nature of cutaneotropic HPVs [5,33,34]. The novel HPVs found in this study were all isolated from healthy individuals from a cohort study of esophageal cancer, in rural Anyang, Henan province, China [15]. Results of general HPV investigation in the Anyang rural population showed very low prevalence of these newly discovered HPVs. Generic primers rather than type-specific primers were used for detecting these novel HPVs so there is a possibility that the prevalence of these novel HPVs was underestimated. 

Systematic positions of HPV 161 to HPV 170, and KC5 were defined by reconstruction of the phylogeny tree based on L1 in 68 HPV types. Although the L1 ORF is the gold standard for defining a PV type, there are many researchers who think that other genes also play important roles in classifying the phylogenetic relationships within the PV family [35,36]. Different genes within the same virus have different evolutionary rates, and different PVs have evolved at different rates [35,37]. In this study, the E1-E2 ORFs were employed in independent phylogenetic analysis to explore possible incongruent topologies. Both early-derived genes (E1-E2) and a late-derived gene (L1) demonstrated that all 11 novel HPVs belong to the γ-PV genus (Figure 1). However, there was incongruence in the topologies of four HPVs (HPV 161, 162, 166, and 168). Values for ML bootstrap support and Bayesian posterior probability were low in some branches inside the γ-PV, and this may be another possible reason for the incongruence between these two independent phylogeny analyses. The identification of phylogenetically representative HPV genome sequences is necessary to fill the gaps in our current knowledge about γ-PV genus.

In conclusion, eleven novel HPV types isolated from normal skin belong to the γ-PV genus. HPV 161, HPV 162, and HPV 166 are a monophyletic group and do not fit into any known species in γ-PV genus, suggesting that these may form a new HPV species in the γ-PV genus. The prevalence of these 11 novel HPVs in the normal population was very low, which strengthens the view that skin HPVs are highly diversified. 

## Supporting Information

Figure S1 **Bayesian tree constructed based on the E1-E2 ORFs.** Bayesian posterior probabilities (lower number, values, 0.90 are not shown). The tree is rooted with selected Mu and Nu species. The analysis involved 68 nucleotide sequences. All positions containing gaps and missing data were eliminated.
(TIF)

Figure S2 **ML tree constructed based on the E1-E2 ORFs.** Numbers on branches are bootstrap support values to the clusters to their right (upper number: ML criterion, values, 50 are not shown). The tree is rooted with selected Mu and Nu species. The analysis involved 68 nucleotide sequences. All positions containing gaps and missing data were eliminated.
(TIF)

Figure S3 **ML tree constructed based on the L1 ORFs.** Numbers on branches are bootstrap support values to the clusters to their right (upper number: ML criterion, values, 50 are not shown). The tree is rooted with selected Mu and Nu species. The analysis involved 68 nucleotide sequences. All positions containing gaps and missing data were eliminated.
(TIF)

Figure S4 **Bayesian tree constructed based on the L1 ORFs.** Bayesian posterior probabilities (lower number, values, 0.90 are not shown). The tree is rooted with selected Mu and Nu species. The analysis involved 68 nucleotide sequences. All positions containing gaps and missing data were eliminated.
(TIF)

Table S1 Positions* of Zinc-binding domains of E6 and E7 ORFs, LxCxE motif of E7 ORF, ATP binding site of the ATP-dependent helicase (GPPDTGKS), and E2-binding site motifs.
(DOCX)

Table S2 **Primers and clone information about the 11 novel HPV types.**
(DOCX)
