# Correction: Identification and Characterization of Eleven Novel Human Gamma-Papillomavirus Isolates from Healthy Skin, Found at Low Frequency in a Normal Population

**DOI:** 10.1371/annotation/974c14e0-9cd1-4600-8259-abdc7927a318

**Published:** 2013-12-13

**Authors:** Jingjing Li, YaQi Pan, QiuJu Deng, Hong Cai, Yang Ke

Figure 1 is incorrect. Please see the correct Figure 1 here: http://plosone.org/corrections/pone.0077116.g001.cn.tif

