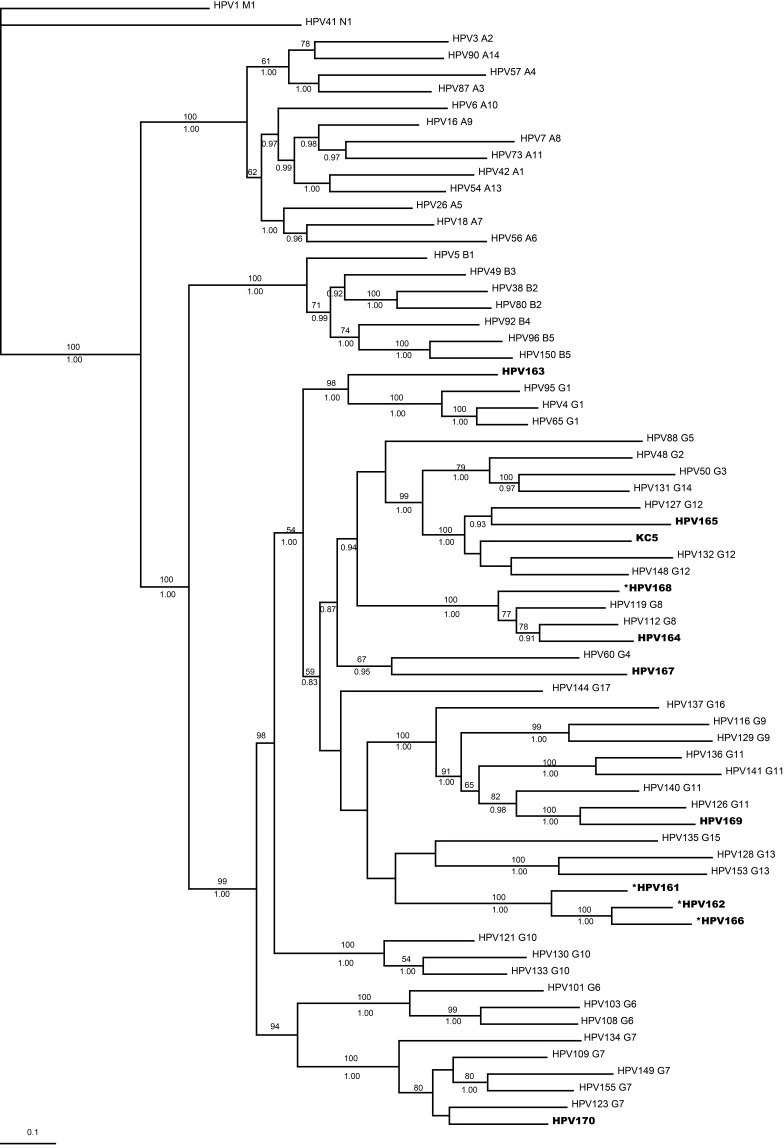# Correction: Identification and Characterization of Eleven Novel Human Gamma-Papillomavirus Isolates from Healthy Skin, Found at Low Frequency in a Normal Population

**DOI:** 10.1371/annotation/fa94362e-5639-42d9-b17b-f89c51c4e9bb

**Published:** 2014-01-06

**Authors:** Jingjing Li, YaQi Pan, QiuJu Deng, Hong Cai, Yang Ke

Figure 2 is incorrect. Please see the correct Figure 2 here: 

**Figure:**