# Supplementary figures and images for: Identification and Characterization of Eleven Novel Human Gamma-Papillomavirus Isolates from Healthy Skin, Found at Low Frequency in a Normal Population
Source: PLoS One. 2013 Oct 14;8(10):e77116. doi: 10.1371/journal.pone.0077116 (PMC3796542; doi:10.1371/journal.pone.0077116)

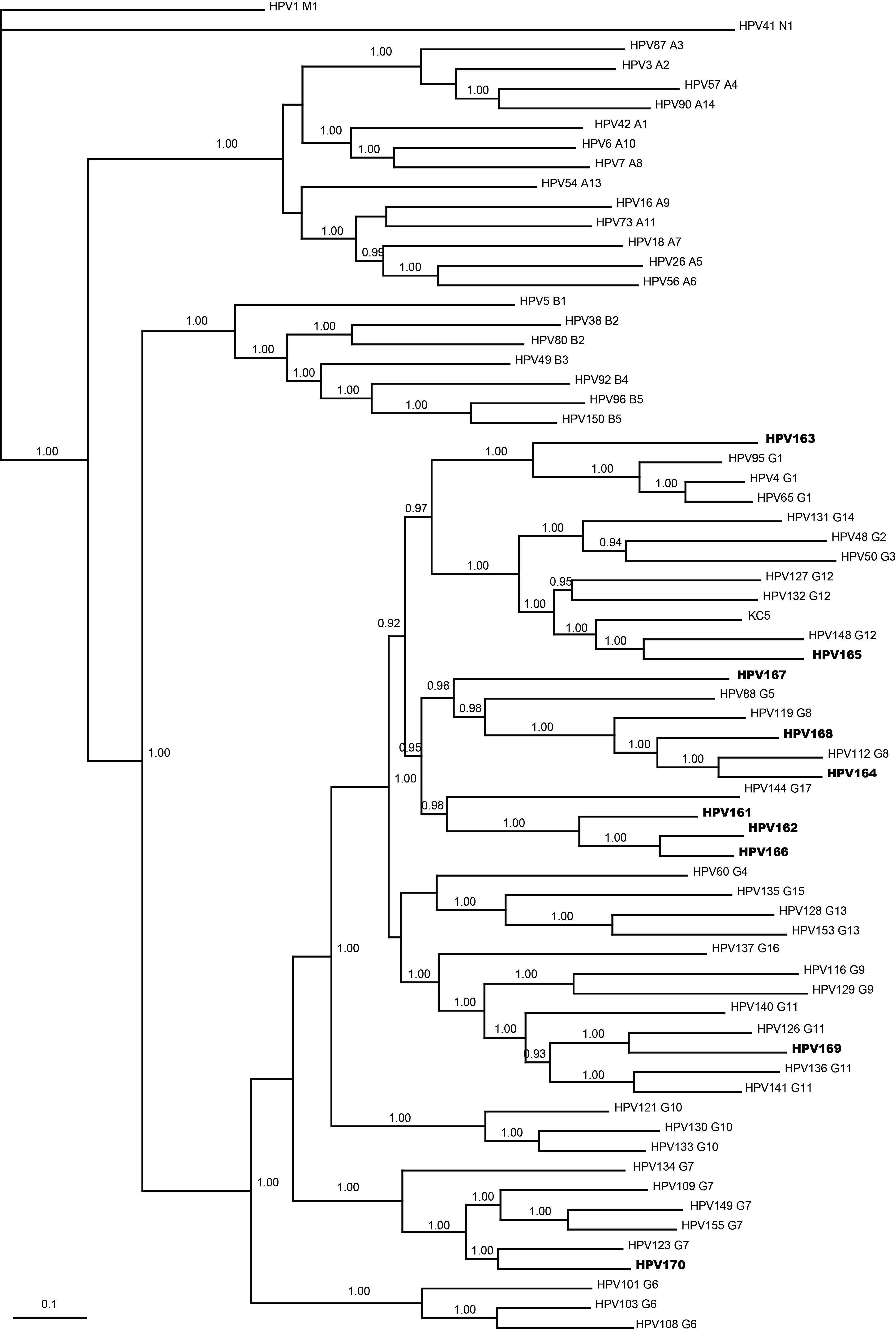

Supplement: Figure S1 — Bayesian tree constructed based on the E1-E2 ORFs. Bayesian posterior probabilities (lower number, values, 0.90 are not shown). The tree is rooted with selected Mu and Nu species. The analysis involved 68 nucleotide sequences. All positions containing gaps and missing data were eliminated. (TIF) [file pone.0077116.s001.tif]

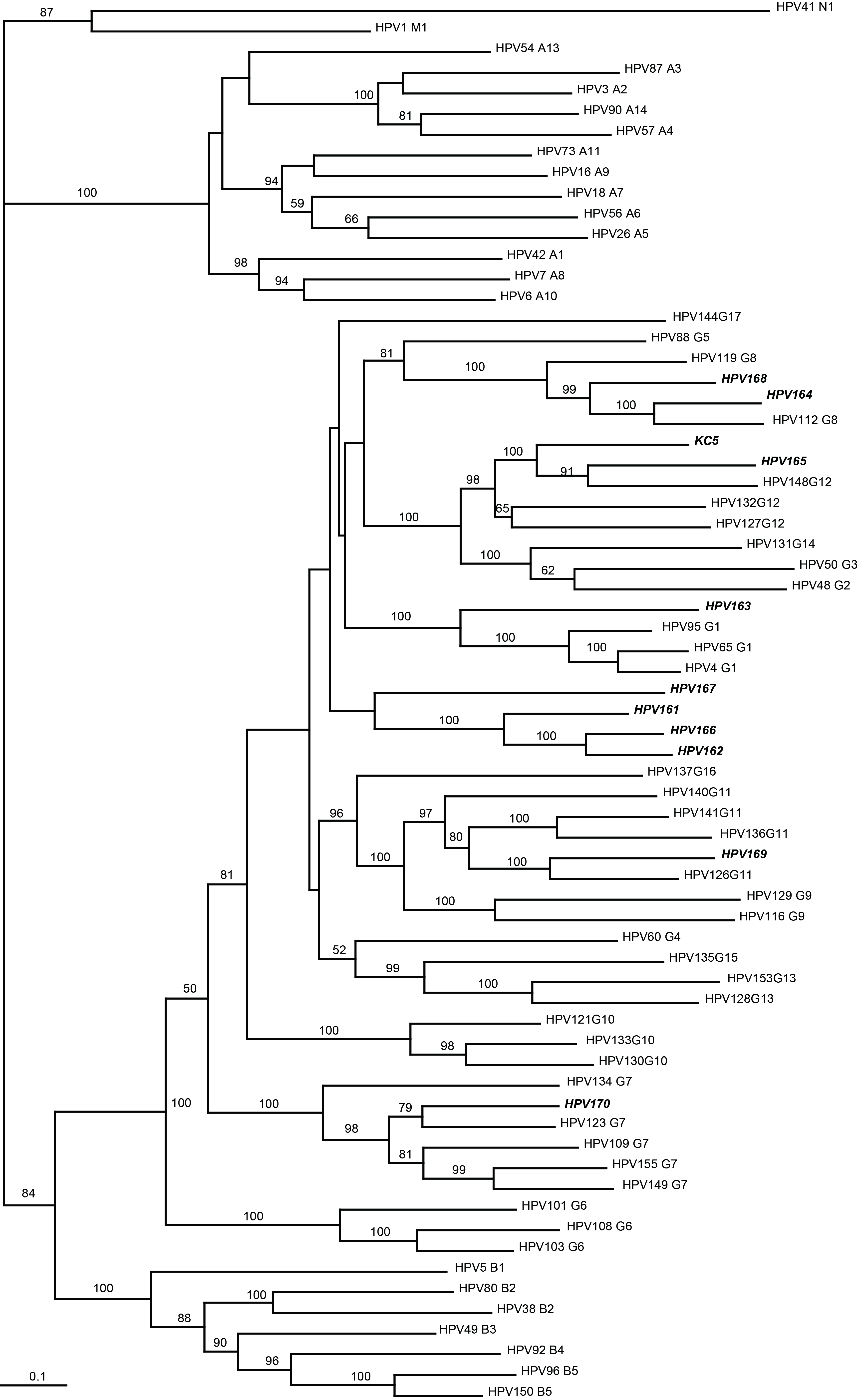

Supplement: Figure S2 — ML tree constructed based on the E1-E2 ORFs. Numbers on branches are bootstrap support values to the clusters to their right (upper number: ML criterion, values, 50 are not shown). The tree is rooted with selected Mu and Nu species. The analysis involved 68 nucleotide sequences. All positions containing gaps and missing data were eliminated. (TIF) [file pone.0077116.s002.tif]

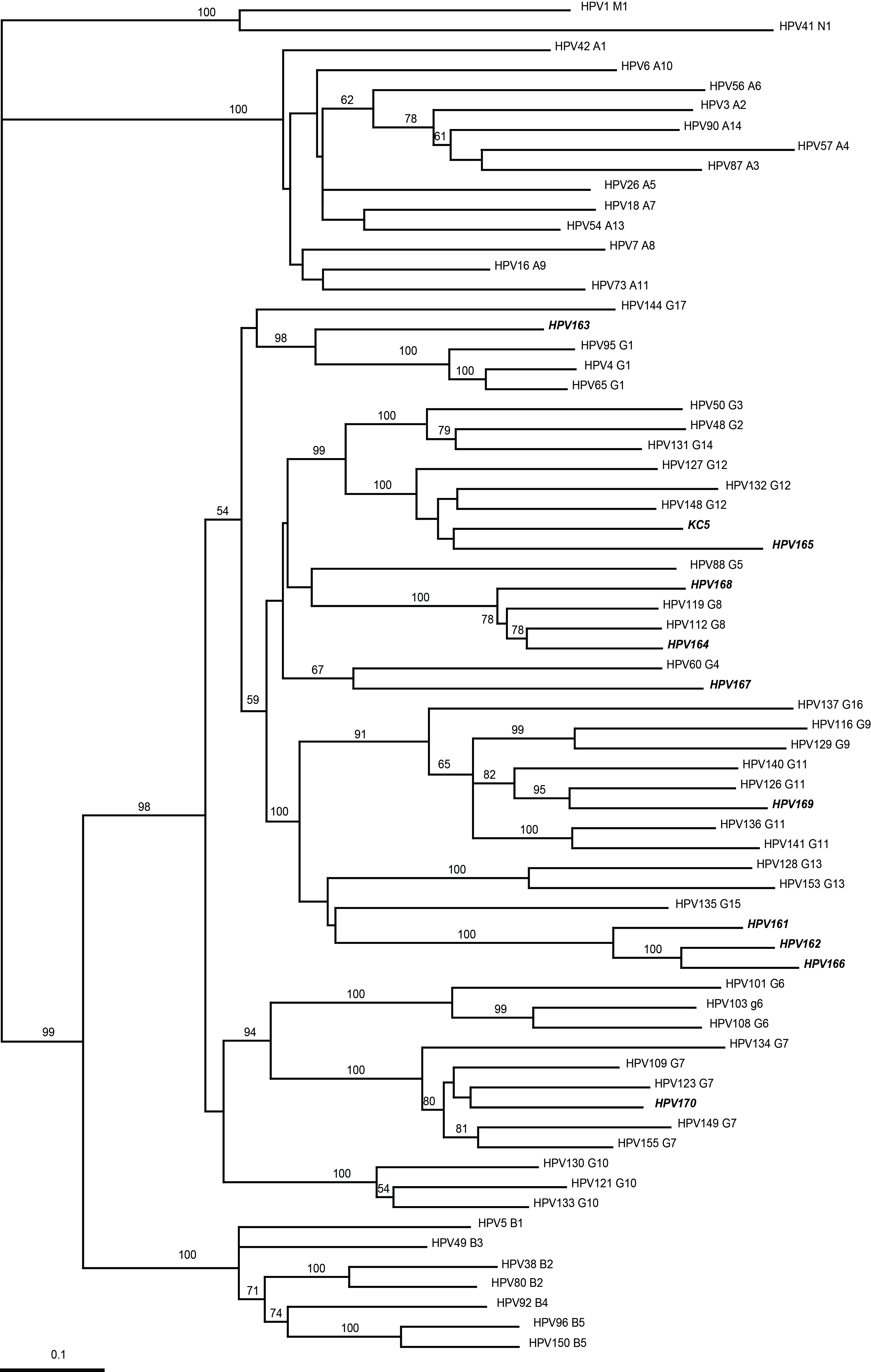

Supplement: Figure S3 — ML tree constructed based on the L1 ORFs. Numbers on branches are bootstrap support values to the clusters to their right (upper number: ML criterion, values, 50 are not shown). The tree is rooted with selected Mu and Nu species. The analysis involved 68 nucleotide sequences. All positions containing gaps and missing data were eliminated. (TIF) [file pone.0077116.s003.tif]

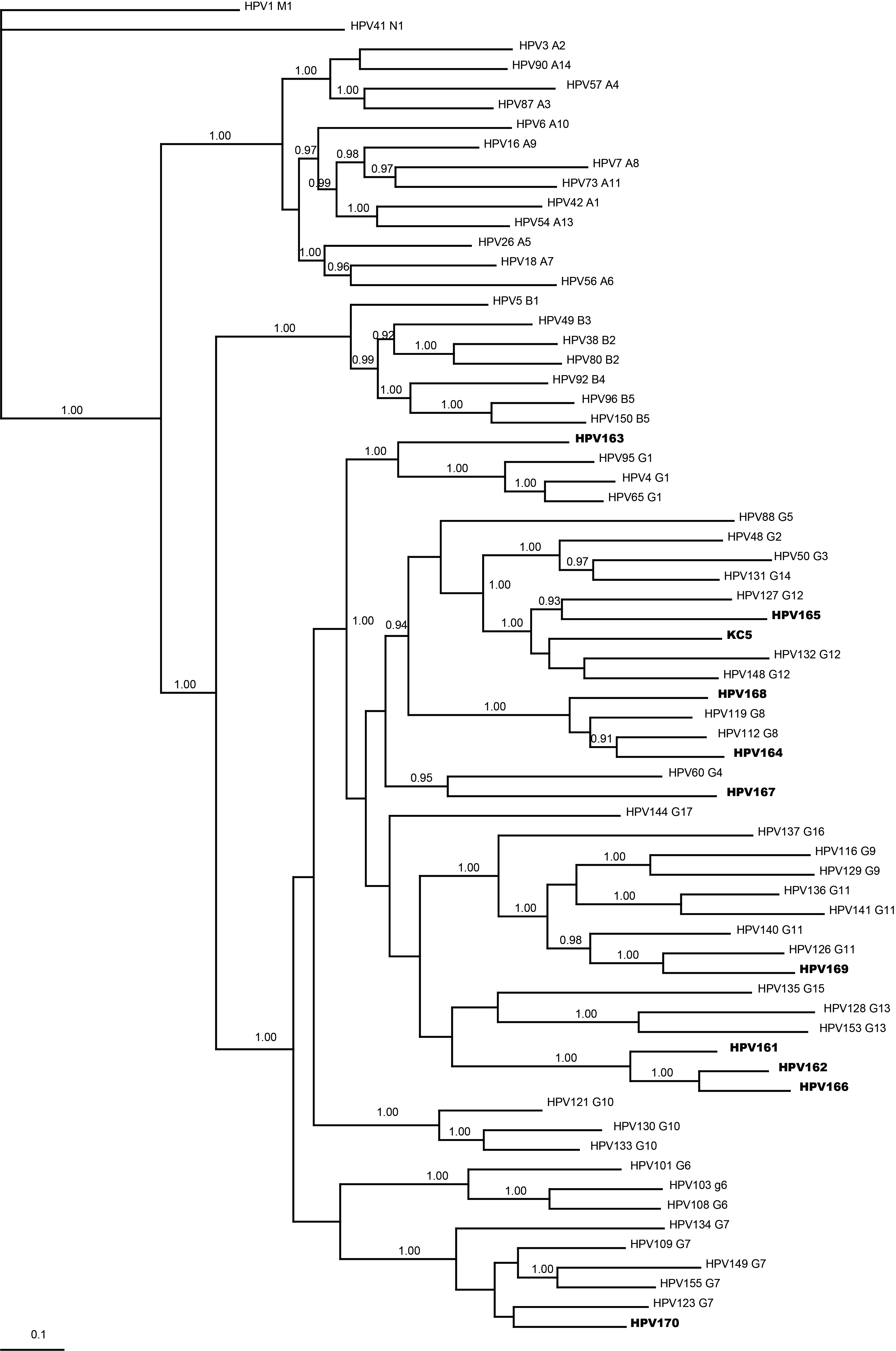

Supplement: Figure S4 — Bayesian tree constructed based on the L1 ORFs. Bayesian posterior probabilities (lower number, values, 0.90 are not shown). The tree is rooted with selected Mu and Nu species. The analysis involved 68 nucleotide sequences. All positions containing gaps and missing data were eliminated. (TIF) [file pone.0077116.s004.tif]
