# Supplementary material for: Identification and Characterization of Eleven Novel Human Gamma-Papillomavirus Isolates from Healthy Skin, Found at Low Frequency in a Normal Population
Source: PLoS One. 2013 Oct 14;8(10):e77116. doi: 10.1371/journal.pone.0077116 (PMC3796542; doi:10.1371/journal.pone.0077116)
Supplement: Table S1 — Positions* of Zinc-binding domains of E6 and E7 ORFs, LxCxE motif of E7 ORF, ATP binding site of the ATP-dependent helicase (GPPDTGKS), and E2-binding site motifs. (DOCX) [file pone.0077116.s005.docx]

**Table S1** Positions* of Zinc-binding domains of E6 and E7 ORFs, LxCxE motif of E7 ORF, ATP binding site of the ATP-dependent helicase (GPPDTGKS), and E2-binding site motifs.

| **HPV type** | **Nucleotide positions of motifs on the genes respectively** | | | | |
| --- | --- | --- | --- | --- | --- |
|  | **zinc-binding domains (E6)** | **zinc-binding domains (E7)** | **LxCxE motif (E7)** | **ATP-dependent helicase (E1)** | **E2 binding site (ACC(N)6GGT) (URR)‡** |
| **HPV 161** | 27-63/100-136 | 50-86 | 22-26 | 432-439 (GPPDTGKS) | 296-307/414-425 **(2)** |
| **HPV 162** | 27-63/100-136 | 54-90 | 24-28 | 435-442 (GPPDTGKS) | 246-257/287-298/407-418/478-489 **(4)** |
| **HPV 163** | 30-66/103-139 | 52-89 | - | 436-443 (GPPDTGKS) | 335-346 **(1)** |
| **HPV 164** | 27-63/100-136 | 50-86 | - | 429-436 (GPPD**S**GKS)† | 177-188/218-228/341-352/411-422 **(4)** |
| **HPV 165** | 26-62/99-135 | 46-82 | 22-26 | 438-445 (GPPDTGKS) | 175-186/328-339/398-409 **(3)** |
| **HPV 166** | 27-63/100-136 | 54-90 | 24-28 | 435-442 (GPPDTGKS) | 239-250/280-291/392-403/463-474 **(4)** |
| **HPV 167** | 27-63/100-135 | 51-87 | 22-26 | 431-438 (GPPDTGKS) | 272-284/452-463 **(2)** |
| **HPV 168** | 27-63/100-136 | 50-86 | - | 429-436 (GPPD**S**GKS)† | 185-196/226-237/348-359/417-428 **(4)** |
| **HPV 169** | 27-63/100-136 | 52-88 | 26-30 | 433-440 (G**V**PD**S**GKS)† | 184-195/216-227/285-296/325-336/414-425/485-496 **(6)** |
| **HPV 170** | 25-61/98-134 | 50-86 | - | 446-453 (GPP**N**TGKS)† | 10-21/302-313/460-471/534-545 **(4)** |
| **KC5** | 26-62/99-135 | 46-82 | - | 426-433 (GPPDTGKS) | 217-228/260-271/370-381 **(3)** |

* Positions is refer to the positions of motifs on the genes (E6, E7, E1) and the upstream regulatory region (URR), but not refer to the position of the genome.

† Amino acid mutation sites were indicated with bold.

‡ E2 binding sites were from 2 to 6 in the URR of each novel HPVs.
