# Supplementary material for: Identification and Characterization of Eleven Novel Human Gamma-Papillomavirus Isolates from Healthy Skin, Found at Low Frequency in a Normal Population
Source: PLoS One. 2013 Oct 14;8(10):e77116. doi: 10.1371/journal.pone.0077116 (PMC3796542; doi:10.1371/journal.pone.0077116)
Supplement: Table S2 — Primers and clone information about the 11 novel HPV types. (DOCX) [file pone.0077116.s006.docx]

**Table S2** Primers and clone information about the 11 novel HPV types

| **Clone** | **PCR Taq** | **Amplicons** | **Overlap** | **Whole genome length** | **Primer1 (5'-3')** | **Primer2 (5'-3')** | **Method** | **Vector** |
| --- | --- | --- | --- | --- | --- | --- | --- | --- |
| **HPV161** | NEB LA taq | 1 | none | 7238 bp | GACGTGCTTAAACTAAGGTCA | CTTACCTGAAACAACTGAAAG | TA | pCR-XL-TOPO^®^ |
| **HPV162** | NEB LA taq | 1 | none | 7214 bp | TACCGGGAGTGGTACAGCTATTG | CAAAGGCTCACAGCCAGAAAC | TA | pCR-XL-TOPO^®^ |
| **HPV163** | NEB LA taq | 1 | none | 7233 bp | AACCGAAAATGATTCAATGTG | GCAGTGAACTTATCTTACCTT | TA | pCR-XL-TOPO^®^ |
| **HPV164** | NEB LA taq | 1 | none | 7233 bp | AACAATAATAGTTGCCAACAA | AAGGAAATCCAAATACCACTC | TA | pCR-XL-TOPO^®^ |
| **HPV165** | NEB LA taq | 1 | none | 7129 bp | TCCTCTGATTGTTGTCTACAA | TAGTAACAGACATTACCGACG | TA | pCR-XL-TOPO^®^ |
| **HPV166** | NEB LA taq | 1 | none | 7212 bp | TCTGTGGTCCTGAAGTAATAA | TAAGATGTTATCGTCTTGACAG | TA | pCR-XL-TOPO^®^ |
| **HPV167** | **NEB phsuion Enzyme** | 2 | L2/LCR region | 7228 bp |  |  | - | pJET1.2/blunt |
|  |  | part1 | 951-960/4495-4514 | 3564 | TGTCAAGAACCGGGAATCTC | GATGAAAGCAACCAGGGAAA | blunt |  |
|  |  | part2 |  | 3694 | TGCTTTCATCCACTTCACCA | GAGATTCCCGGTTCTTGACA | blunt |  |
| **HPV168** | **NEB phsuion Enzyme** | 2 | L2/LCR region | 7126 bp | - | - | - | pJET1.2/blunt |
|  |  | part1 | 4305-4322 | 4322 bp | TGGGGTCGTTATTTCCATGT | GATCAGCAACACCTGCTTCA | blunt |  |
|  |  | part2 |  | 3130 bp | AAGCAGGTGTTGCTGATCCT | TACCACTCCCGGTACAAAGG | blunt |  |
| **HPV169** | NEB LA taq | 1 | none | 7252 bp | GTACCACTTTTGTTACTGGCT | TGCCAATACGTGATTCAGTTA | TA | pCR-XL-TOPO^®^ |
| **HPV170** | NEB LA taq | 1 | none | 7417 bp | AGCATCTGCAAACAAACACTT | TGCAAGCCTATTTATGTAGCC | TA | pCR-XL-TOPO^®^ |
| **KC5** | NEB LA taq | 1 | none | 7143 bp | CAACAATCATCTGACTAGCTA | GCAACAATAACGATAGAGGAA | TA | pCR-XL-TOPO^®^ |
